# High-Frequency Electrical Stimulation Can Be a Complementary Therapy to Promote Nerve Regeneration in Diabetic Rats

**DOI:** 10.1371/journal.pone.0079078

**Published:** 2013-11-12

**Authors:** Chia-Hong Kao, Jia-Jin J. Chen, Yuan-Man Hsu, Da-Tian Bau, Chun-Hsu Yao, Yueh-Sheng Chen

**Affiliations:** 1 Lab of Biomaterials, School of Chinese Medicine, China Medical University, Taichung, Taiwan; 2 Department of Chinese Medicine, Taipei Medical University Hospital, Taipei, Taiwan; 3 Department of Biomedical Engineering, National Cheng Kung University, Tainan, Taiwan; 4 Department of Biological Science and Technology, China Medical University, Taichung, Taiwan; 5 Terry Fox Cancer Research Lab, China Medical University Hospital, Taichung, Taiwan; 6 Department of Biomedical Imaging and Radiological Science, China Medical University, Taichung, Taiwan; 7 Department of Biomedical Informatics, Asia University, Wufeng District, Taichung, Taiwan; University of Palermo, Italy

## Abstract

The purpose of this study was to evaluate whether 1 mA of percutaneous electrical stimulation (ES) at 0, 2, 20, or 200 Hz augments regeneration between the proximal and distal nerve stumps in streptozotocin diabetic rats. A10-mm gap was made in the diabetic rat sciatic nerve by suturing the stumps into silicone rubber tubes. Normal animals were used as the controls. Starting 1 week after transection, ES was applied between the cathode placed at the distal stump and the anode at the proximal stump every other day for 3 weeks. At 4 weeks after surgery, the normal controls and the groups receiving ES at 20, and 200 Hz had a higher success percentage of regeneration compared to the ES groups at 0 and 2 Hz. In addition, quantitative histology of the successfully regenerated nerves revealed that the groups receiving ES at a higher frequency, especially at 200 Hz, had a more mature structure with more myelinated fibers compared to those in the lower-frequency ES groups. Similarly, electrophysiology in the ES group at 200 Hz showed significantly shorter latency, larger amplitude, larger area of evoked muscle action potentials and faster conduction velocity compared to other groups. Immunohistochemical staining showed that ES at a higher frequency could significantly promote calcitonin gene-related peptide expression in lamina I-II regions in the dorsal horn and recruit a higher number of macrophages in the diabetic distal sciatic nerve. The macrophages were found that they could stimulate the secretion of nerve growth factor, platelet-derived growth factor, and transforming growth factor-β in dissected sciatic nerve segments. The ES at a higher frequency could also increase cutaneous blood flow in the ipsilateral hindpaw to the injury. These results indicated that a high-frequency ES could be necessary to heal severed diabetic peripheral nerve with a long gap to be repaired.

## Introduction

Peripheral diabetic neuropathy leads to irreversible disability through axonal atrophy and progressive loss of axons. Until now, it is still a big challenge of how to promote regeneration of diabetic peripheral nerve. Streptozotocin (STZ)-induced rats typically have been examined in studies of diabetic peripheral nerve regeneration. Results obtained from such studies commonly showed a delay in the onset of regeneration after nerve injury that later also influences the elongation, myelination, and maturation of axonal sprouts [1]. Diabetes can also cause a decrease in nerve conduction and a reduction in nerve blood flow in rats, which are similar to the early neuropathy seen in diabetic patients [2]. It has been reported that electrical stimulation (ES) treatments could be a complementary tool for promoting axonal regeneration, rising local blood flow to support regenerative events, and improving nerve function in diabetic rats [3], [4]. However, most diabetic animals used in the literature have not shown nerve fiber loss in steady state, and it is not satisfactory to use an uninjured nerve or an injury nerve with only a small amount of nerve fiber lesions [5]. To provide essential evidence for the therapeutic potential of the ES treatment, we have successfully demonstrated the beneficial effects of percutaneous ES to long sciatic nerve defects in STZ-rats [6]. Since the capacity of this modality to enhance nerve regeneration could be influenced by the frequency of administered ES, this study was performed to investigate the effects of four different frequencies of ES at 0, 2, 20, and 200 Hz on peripheral nerve regeneration in STZ-rats with a 10-mm nerve gap, which was repaired with a silicone rubber nerve tube. After a 4-week recovery period, nerve regeneration was then assessed by measuring cutaneous blood flow in the hindlimb footpads, calcitonin gene-related peptide (CGRP) in the lumbar spinal cord, macrophage influx in the distal nerve end, as well as electrophysiology and morphology of the regenerated nerve in the bridging conduit. Finally, since the macrophages have been considered a vital role in peripheral nerve regeneration mainly because they are profoundly involved in regulating the expression of neurotrophic factors by releasing interleukin-1 (IL-1) [7], [8], we therefore studied the changes of mRNA levels of fibroblast growth factor (FGF), nerve growth factor (NGF), platelet-derived growth factor (PDGF), and transforming growth factor-β (TGF-β) of rat sciatic nerve segments by adding conditioned media of recombinant rat IL-1β to elucidate mechanisms underlying the observed ES effects at different frequencies.

## Materials and Methods

### Induction of Diabetes

Prior to the beginning of the *in vivo* testing, the protocol was approved by the ethical committee for animal experiments of the China Medical University, Taichung, Taiwan. Diabetes was induced in adult male Sprague-Dawley rats (250–300 g, BioLasco Co, Ltd, Taipei, Taiwan) by tail vein injection of a single 50 mg/kg dose of STZ (Sigma Chemical Co, St Louis, MO). The STZ was solubilized in normal saline immediately before injection. Seven days after STZ injection, serum glucose measurements were determined on all animals with a glucose analyzer (Accu-Chek, Roche, Basel, Switzerland). Animals with an initial blood glucose of 300 mg/dl or greater qualified as diabetic.

### Implantation of Silicone Rubber Chambers

All rats were anesthetized with an inhalational technique (AErrane; Baxter, Deerfield, IL). The right sciatic nerves were severed into proximal and distal segments. The proximal stump was then secured with a single 9–0 nylon suture through the epineurium and the outer wall of a silicone rubber chamber (1.47 mm inner diameter, 1.96 mm outer diameter; Helix Medical, Inc, Carpinteria, CA). The distal stump was secured into the other end of the chamber. Both the proximal and the distal stumps were secured to a depth of 1 mm into the chamber, leaving a 10-mm gap between the stumps. The muscle and skin were closed. All animals were housed in temperature (22°C) and humidity (45%) controlled rooms with 12-hour light cycles. They had access to food and water ad libitum.

### Electrical Stimulation Protocols

The ES treatment protocol was previously reported [9]. In brief, animals were secured in a small cage and their stretched right leg and paw were held in place by rubber tapes. One stainless steel needle electrode (0.35 mm outer diameter, 12 mm length) connected to the negative wick (cathode) of a stimulator (Trio 300; Ito, Tokyo, Japan) was inserted aseptically into the lateral aspect of the knee, and the anode was positioned around the site of the hip joint. The positive and negative stimulating sites were near the proximal and distal ends of the implanted silicone tubes, respectively. The depth of insertion varied from 1 to 1.5 cm according to the thickness of skin and fatty tissues. The stimulation was applied to the animals for 15 minutes every other day beginning a week after the nerve repair, to avoid loosening the suture line which might also cause inflammation. The animals were divided into five groups. Group A (n = 10), the controls, normal animals received empty silicone rubber chambers only. Groups B-E (n = 10 for each group), diabetic animals received a treatment of electrical stimulation of 1 mA at frequencies of 0, 2, 20, and 200 Hz, respectively, after their injured nerves were bridged with the silicone rubber tubes. Cutaneous blood flow in the hindlimb footpad ipsilateral to the injury of the rat was measured with a laser Doppler flowmetry device (wavelength, 780 nm; DRT4; Moor Instruments Ltd., Millwey, Axminster, UK) at various time points: 7 d, 14 d, 21 d, and 28 d after the nerve repair.

### Electrophysiological Techniques

Four weeks after nerve repair, all animals were re-anesthetized and the sciatic nerve exposed. The nerve was given a supramaximal stimulus through a pair of needle electrodes placed directly on the sciatic nerve trunk, 5-mm proximal to the transection site. Latency, amplitude, and area of the evoked muscle action potentials (MAPs) were recorded from the gastrocnemius muscle with microneedle electrodes linked to a computer (Biopac Systems, Inc., Goleta, California). The latency was measured from stimulus to the takeoff of the first negative deflection. The amplitude and the area under the MAP curve from the baseline to the maximal negative peak were calculated. The MAP was then used to calculate the nerve conductive velocity (NCV), which was carried out by placing the recording electrodes in the gastrocnemius muscles and stimulating the sciatic nerve proximally and distally to the silicone rubber conduit. The NCV was then calculated by dividing the distance between the stimulating sites by the difference in latency time.

### Histological Techniques

Immediately after the recording of muscle action potential, all of the rats were perfused transacrdially with 150 ml normal saline followed by 300 ml 4% paraformaldehtde in 0.1 M phosphate buffer, pH 7.4. After perfusion, the L4 spinal cord and the distal stump outside the nerve gap were quickly removed and post-fixed in the same fixative for 3–4 h. Tissue samples were placed overnight in 30% sucrose for cryoprotection at 4°C, followed by embedding in optimal cutting temperature solution. Samples were the kept at −20°C until preparation of 18 µm sections was performed using a cryostat, with samples placed upon poly-L-lysine-coated slide. Immunohistochemistry of frozen sections was carried out using a two-step protocol according to the manufacturer's instructions (Novolink Polymer Detection System, Novocastra). Briefly, frozen sections were required endogenous peroxidase activity was blocked with incubation of the slides in 0.3% H_2_O_2_, and nonspecific binding sites were blocked with Protein Block (RE7102; Novocastra). After serial incubation with rabbit- anti-CGRP polyclonal antibody 1∶1000 (calbiochem, Germany), Post Primary Block (RE7111; Novocastra), and secondary antibody (Novolink Polymer RE7112), the L4 spinal cord sections were developed in diaminobenzidine solution under a microscope and counterstained with hematoxylin. Similar protocols were applied in the sections from the distal stump except they were incubated with anti-rat CD68 1:100 (AbD Serotec, Kidlington, UK). Sciatic nerve sections were taken from the middle regions of the regenerated nerve in the chamber. After the fixation, the nerve tissue was post-fixed in 0.5% osmium tetroxide, dehydrated, and embedded in Spurr’s resin. The tissue was then cut to 2-µm thickness by using a microtome (Leica EM UC6, Leica Biosystems, Mount Waverley, Australia) with a diamond knife, stained with toluidine blue.

### Changes in mRNA Levels of FGF, NGF, PDGF, and TGF-β of Rat Sciatic Nerve Segments Conditioned by IL-1β

Sciatic nerve segments (3 cm) of adult Sprague-Dawley rats were cultured in 1 ml Dulbecco’s modified Eagle’s medium supplemented with 10% fetal calf serum. After three days culture, 200 ng/ml of medium conditioned by recombinant rat IL-1β (PeproTech, Rocky Hill, NJ) was added. Total RNAs were extracted from nerve tissues with TRIzol reagent and the amount of RNA estimated by spectrophotometry at 260 nm. For real-time (RT)-PCR analysis, two-step RT-PCR was carried out using a high-capacity cDNA reverse transcription kit (Applied Biosystems, USA), and a 16S rRNA gene PCR assay was used as a housekeeping gene control assay. The reactions were performed in 20 µl (total volume) mixtures containing primers at a concentration of 400 nM. The reaction conditions consisted of 2 min at 50°C, 10 min at 95°C, then 40 cycles of 15 s at 95°C, followed by 1 min at 60°C. Melting curve analysis was used to determine the PCR specificity and was performed using 80 10-s cycles, with the first cycle at 60°C and the temperature increasing by 0.5°C for each succeeding cycle. All reactions were carried out in triplicate from three cultures. Each assay was run on an Applied Biosystems 7300 Real-Time PCR system. The threshold cycle (Ct) is defined as the fractional cycle number at which the fluorescence passes the fixed threshold data, and was determined using the default threshold settings. Relative quantification of mRNA expression was calculated using the 2-ΔΔCt method (Applied Biosystems User Bulletin N°2 (P/N 4303859)). Data were presented as the relative expression of target mRNA, normalized with respect to GAPDH mRNA and relative to a calibrator sample that was collected at 0 min of infection. The primers used in this study are shown in Table 1.

**Table 1:** PCR primers.

rat NGF-F	GTGGACCCCAAACTGTTTAAGAA
rat NGF-R	AGTCTAAATCCAGAGTGTCCGAAGA
rat FGF-F	ACGGCGTCCGGGAGAA
rat FGF-R	AGGTACCGGTTCGCACACA
rat PDGFa-F	AGGATGCCTTGGAGACAAACC
rat PDGFa-R	TCAATACTTCTCTTCCTGCGAATG
rat TGFb-F	CACCGGAGAGCCCTGGATA
rat TGFb-R	TCCAACCCAGGTCCTTCCTA
rat-GAPDH-F	GGTGGACCTCATGGCCTACA
Rat-GAPDH-R	CAGCAACTGAGGGCCTCTCT

### Image Analysis

All tissue samples were observed under an optical microscope (Olympus IX70; Olympus Optical Co, Ltd, Tokyo, Japan) with an image analyzer system (Image-Pro Lite; Media Cybernetics, Silver Spring, MD). CGRP-immunoreactivity (IR) in dorsal horn in the lumbar spinal cord was detected by immunohistochemistry as described previously [10]. The immuno-products were confirmed positive-labeled if their density level was over five times background levels. Under a 400× magnification, the ratio of area occupied by positive CGRP-IR in dorsal horn ipsilateral to the injury following neurorrhaphy relative to the lumbar spinal cord was measured. The number of neural components in each nerve section was also counted. As counting the myelinated axons, at least 30 to 50 percent of the sciatic nerve section area randomly selected from each nerve specimen at a magnification of 400× was observed. The axon counts were extrapolated by using the area algorithm to estimate the total number of axons for each nerve. Similarly, macrophages were counted in each nerve section at the distal stump and the density of macrophages was obtained by dividing the macrophage counts by the total nerve areas.

### Statistical Analysis

For the statistical analysis of immunohistochemical, morphometric, and electrophysiological measurements of regenerated nerves, data were collected by the same observer and expressed as mean ± standard deviation, and comparisons between groups were made by the 1-way analysis of variance (SAS 8.02). The Tukey test was then used as a post hoc test. Statistical significance was set at *P*<0.05.

## Results

The cutaneous blood flow in the ipsilateral hindpaw to the injury in response to ES at four frequencies including 0, 2, 20, and 200 Hz and the normal control at different time points measured is shown in Table 2. Mean blood flow in un-stimulated animals in the control group at each of the four time points measured was about 300 perfusion units, which was significantly larger than that in all of the four ES-stimulated diabetic groups (p<0.05). Among stimulated diabetic animals, their blood flows were dramatically increased as elevating the ES frequency. It was noted that the ES at 200 Hz triggered a higher increase in blood flow compared to that observed at 0, 2, and 20 Hz, especially at 7 d of post-surgery that all of the differences reached the significance at p<0.05. There was no statistically difference between blood flow changes at 2 and 20 Hz at each of the time points measured.

**Table 2:** Blood flow to the ipsilateral hindpaw in response to ES at 0, 2, 20, and 200

Perfusion unit				
Groups (n)	7 d	14 d	21 d	28 d
**A Control (10)**	332±27^+^ § ¶ ^#^	323±18^+^ § ¶ ^#^	335±42^+^ § ¶ ^#^	292±6^+^ § ¶ ^#^
**B 0 Hz (10)**	122±10* § ¶ ^#^	118±13* § ¶ ^#^	122±8* § ¶ ^#^	124±15* § ¶ ^#^
**C 2 Hz (10)**	141±6* ^+#^	144±7* ^+#^	142±8* ^+#^	143±13* +
**D 20 Hz(10)**	142±10* ^+#^	147±9* +	148±15* +	143±10* +
**E 200 Hz(10)**	154±16* ^+^ § ¶	165±15* ^+^ §	161±12* ^+^ §	149±12* +

Each data point represents the mean ± SEM (n = 10).

* p<0.05 compared with group A.

+ p<0.05 compared with group B.

§ p<0.05 compared with group C.

¶ p<0.05 compared with group D.

# p<0.05 compared with group E.

Similar electrophysiological patterns during the entire assessment period were seen among the ES-treated animals and the controls. It was noted that larger NCVs, amplitudes, and areas of the MAPs and shorter latencies were seen as the frequency of ES was increased, where the differences of all of these parameters between the ES groups at 0 Hz and 200 Hz reached the significance at p<0.05 (Table 3).

**Table 3:** Analysis of electrophysiological recordings of CMAPs for the (a) conductive velocity in nerves supplying gastrocnemius muscle; (b) peak amplitude; (c) area; (d) latency four weeks postoperatively.

Electrophysiological Analysis				
Groups (n)	NCV (m/s)	Latency (ms)	Amplitude (mV)	MAP area (mVms)
**A Control (6)**	31.5±4.6^+^ §	1.2±0.1^+^ § ¶	6.6±1.0#	7.7±1.2#
**B 0 Hz (4)**	26.1±2.6* ¶ ^#^	1.5±0.3* #	6.6±1.9#	7.8±1.6#
**C 2 Hz (4)**	27.3±2.4* #	1.4±0.2* #	7.5±1.9#	8.7±2.2#
**D 20 Hz(5)**	29.5±1.0^+#^	1.4±0.1* #	7.9±2.2#	9.6±1.9
**E 200 Hz(6)**	33.2±1.6^+^ § ¶	1.2±0.1^+^ § ¶	9.6±2.7* ^+^ § ¶	11.4±3.5* ^+^ §

Each data point represents the mean ± SEM.

* p<0.05 compared with group A.

+ p<0.05 compared with group B.

§ p<0.05 compared with group C.

¶ p<0.05 compared with group D.

# p<0.05 compared with group E.

**Figure 1:**
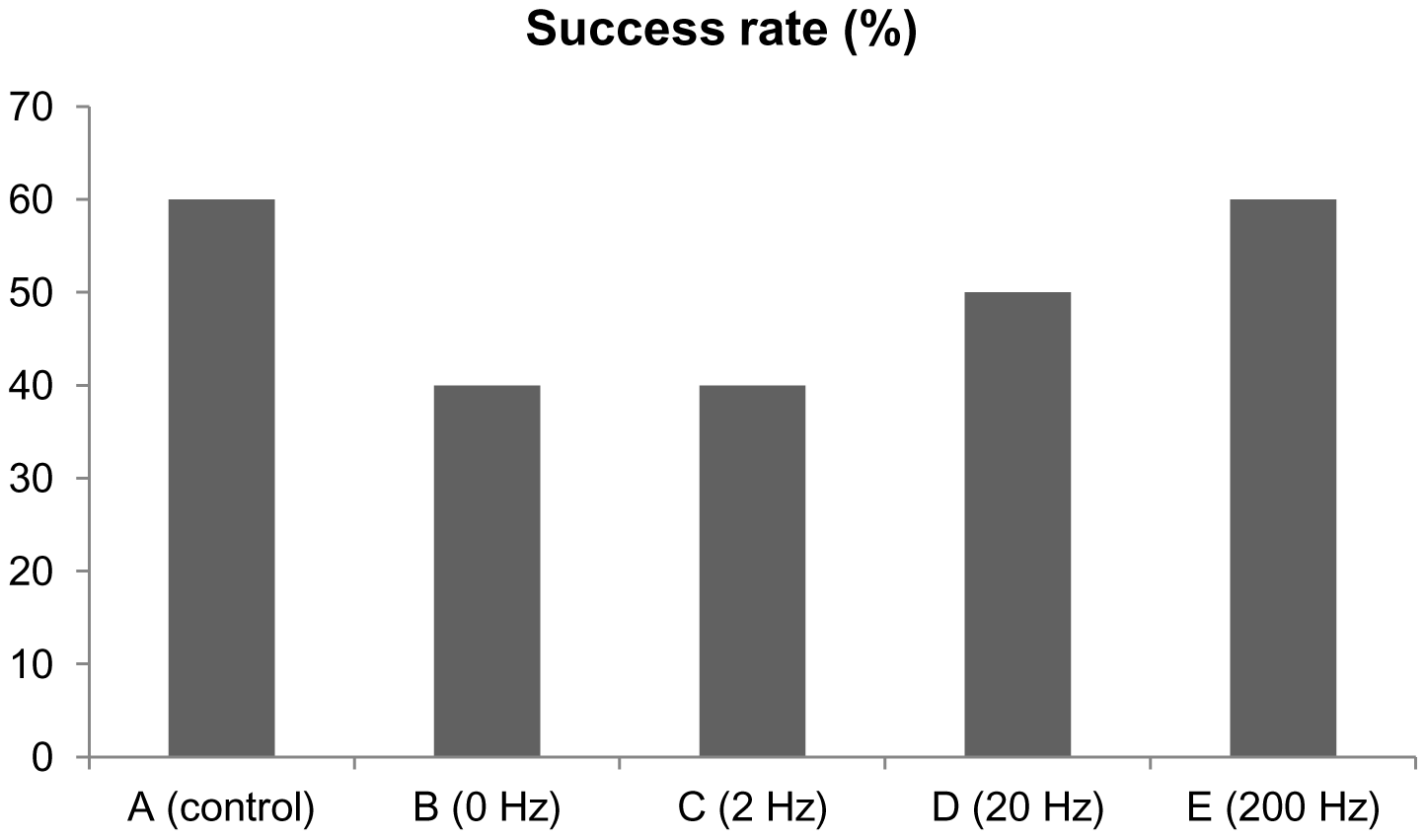
Success rates of regenerated nerves across the 10-mm gaps.

**Figure 2:**
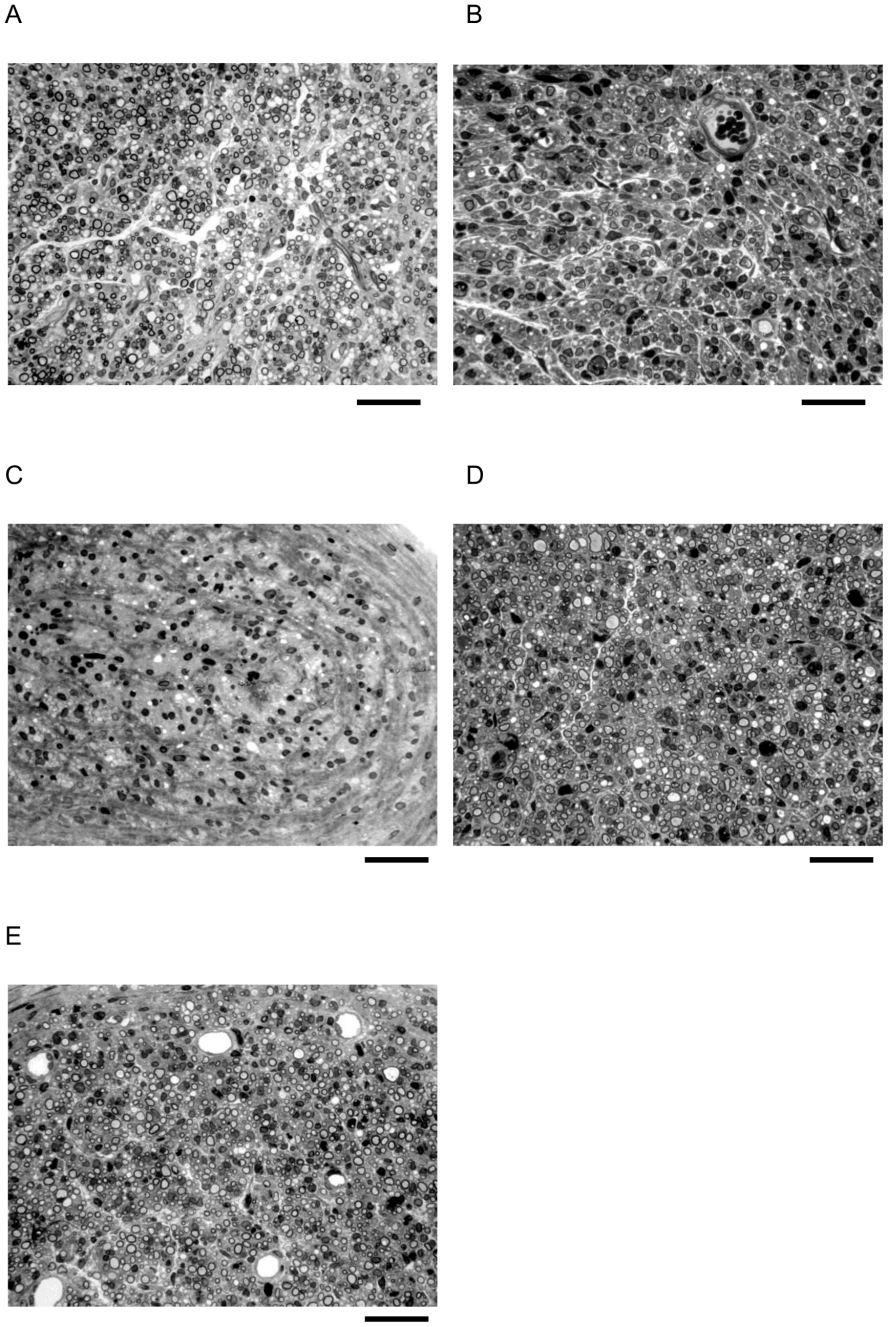
Temporal profile of morphologic changes of a normal control from an animal of (a) group A, and the diabetic sciatic nerves after ES treatment four weeks postoperatively from (b) group B (0 Hz), (c) group C (2 Hz), (d) group D (20 Hz), and (e) group E (200 Hz). The nerves in groups B and C showed less mature structures with mostly Schwann cells and few myelinated axons dispersed randomly over the endoneurium. It was noted that the nerves in groups A, D, and E had numerous myelinated axons distributed in a more compact neural connective tissue structure. Bar = 30 µm.

To determine whether different frequencies of ES could affect nerve regeneration, success rate of cable formation in the silicone rubber conduits was measured. A proportion (6 of 10 rats) formed regenerative outgrowth in the normal controls (Figure 1). In comparison, 40, 40, 50, and 60% showed regenerated nerve cables in the ES groups of 0, 2, 20, and 200 Hz, respectively. In the successfully regenerated cables, it appears that ES-treated diabetic animals at 0 Hz had a significant reduction in the number of myelinated axons even after 4 weeks of regeneration (Table 4). Only one in the four regenerated cables in the this group showed a myelinated axon count more than 1000 from the midportion of the cable, whereas the other three cables were poorly formed, contained much fewer myelinated axons and mostly Schwann cells and blood vessels only (Figure 2). Similar results were noted in the ES-group at 2 Hz that most of the regenerated nerves were immature with few myelinated axons. In comparison, two of five and three of six regenerated cables in the ES group at 20 and 200 Hz had more than 1000 myelinated axons which were similar to those seen in the normal controls, implying that the elevated frequency of ES could exert growth-promoting capability on regenerating diabetic nerve fibers. Considering the ES-treated samples examined, the findings identified a nonsignificant trend toward a rise in axon penetration into the regenerated cables as increasing the ES frequency.

**Table 4:** Morphometric analysis of axonal regeneration and remyelination.

Myelinated axon count (#)					
Animal #	A (control)	B (0 Hz)	C (2 Hz)	D (20 Hz)	E (200 Hz)
**1**	1547	–	–	430	–
**2**	1352	225	–	–	–
**3**	–	–	–	–	5
**4**	–	700	–	–	1008
**5**	2012	–	–	–	–
**6**	1911	–	1674	2251	13
**7**	–	–	–	–	68
**8**	–	–	32	240	4110
**9**	2184	1607	38	2463	1026
**10**	1596	126	18	8	–
**Mean**	1767	665	440	1078	1038
**S.D.**	318	677	822	1179	1581

It was noted that the number of myelinated axons in the ES-treated groups at 20 and 200 Hz was much greater than that in the ES groups at 0 and 2 Hz.

**Figure 3:**
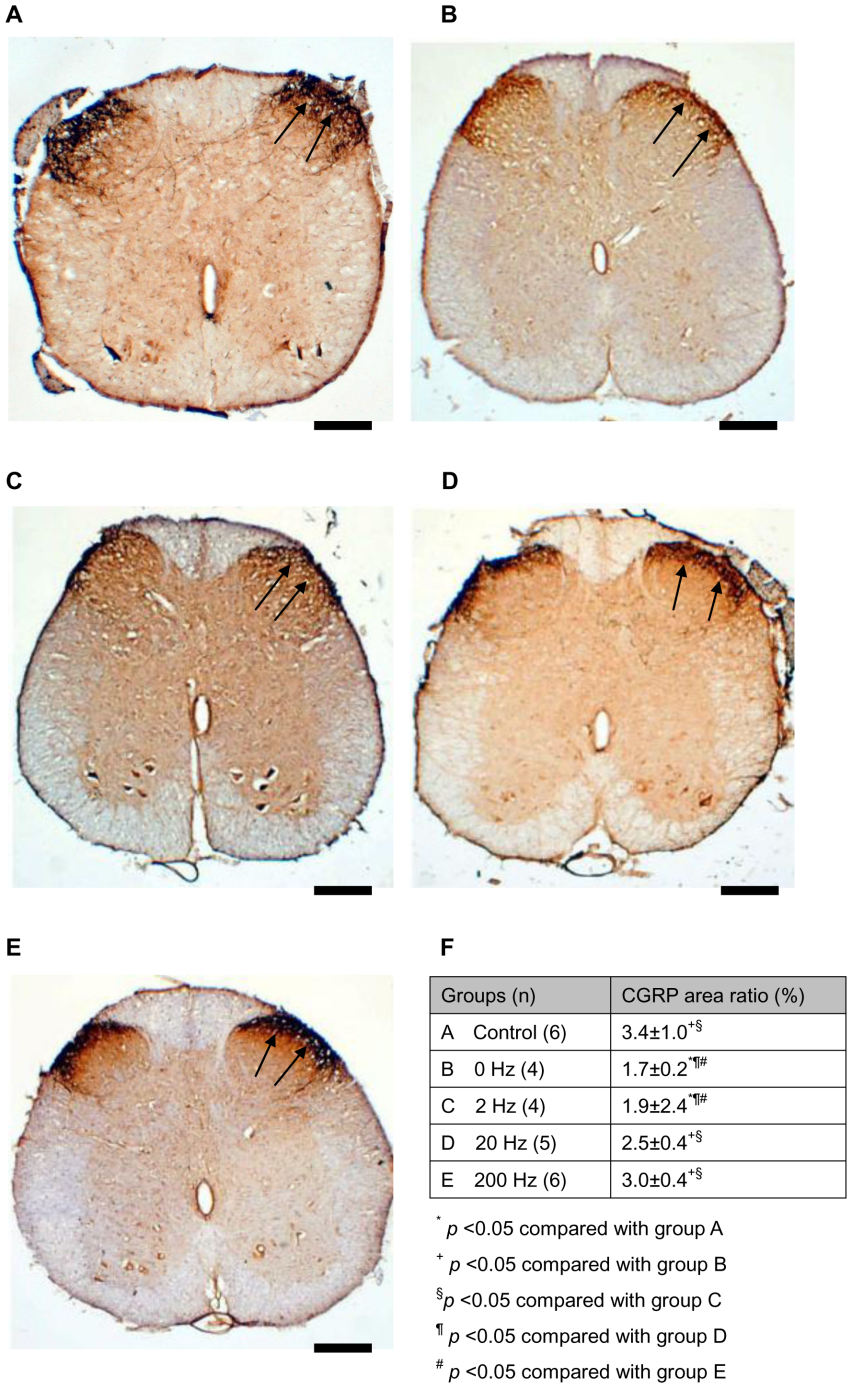
Photomicrographs demonstrating CGRP-IR (arrows) in dorsal horn in the lumbar spinal cord after injury of a normal control from an animal of (a) group A, and the diabetic sciatic nerves after ES treatment from (b) group B (0 Hz), (c) group C (2 Hz), (d) group D (20 Hz), and (e) group E (200 Hz). (f) Note the significantly increased CGRP-IR area ratios in diabetic animals receiving a higher frequency of ES. Each data point represents the mean ± SEM. Bar = 200 µm.

Immunohistochemical staining showed that CGRP-labeled fibers were seen in the area of lamina III–V and lamina I–II regions in the dorsal horn ipsilateral to the injury in all of the rats (Figure 3). As compared to the normal controls, the ratio of area occupied by positive CGRP-IR was dramatically decreased in the diabetic animals even they had received the ES treatment. However, it was noted that higher-frequency ES groups had a higher ratio of area occupied by positive CGRP-IR compared to lower-frequency ES-treated animals. Among those stimulated animals, ES at 20 and 200 Hz had a significantly higher ratio of CGRP-IR area compared to ES at 0 and 2 Hz (p<0.05).

**Figure 4:**
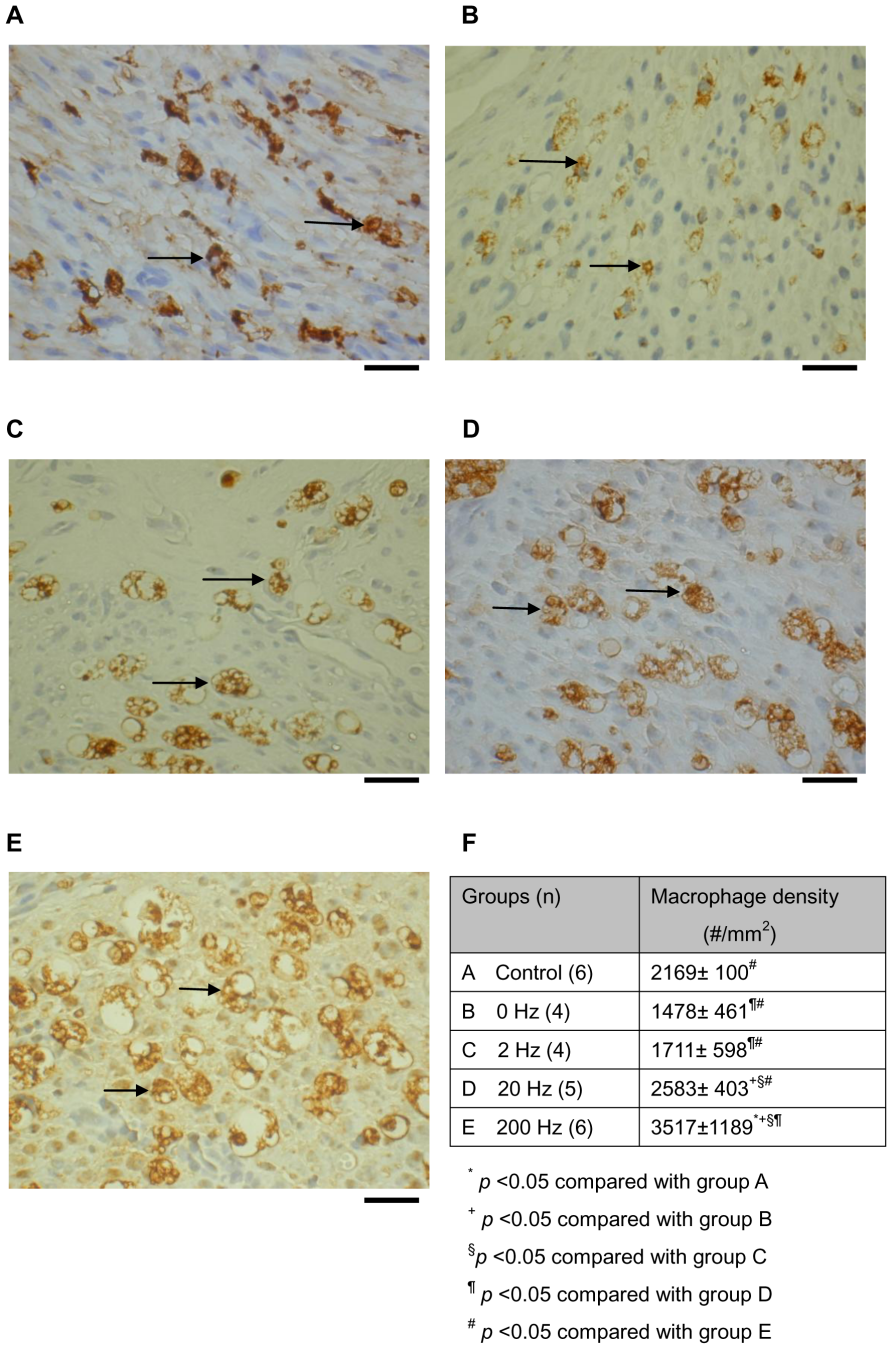
Photomicrographs demonstrating anti-rat CD68 immunoreactivity in macrophages (arrows) from cross sections of distal nerve cables of (a) group A, (b) group B, (c) group C, (d) group D, (e) and group E. (f) Note the significantly increased density of macrophages in diabetic animals receiving a higher frequency of ES. Each data point represents the mean ± SEM. Bar = 25 µm.

**Figure 5:**
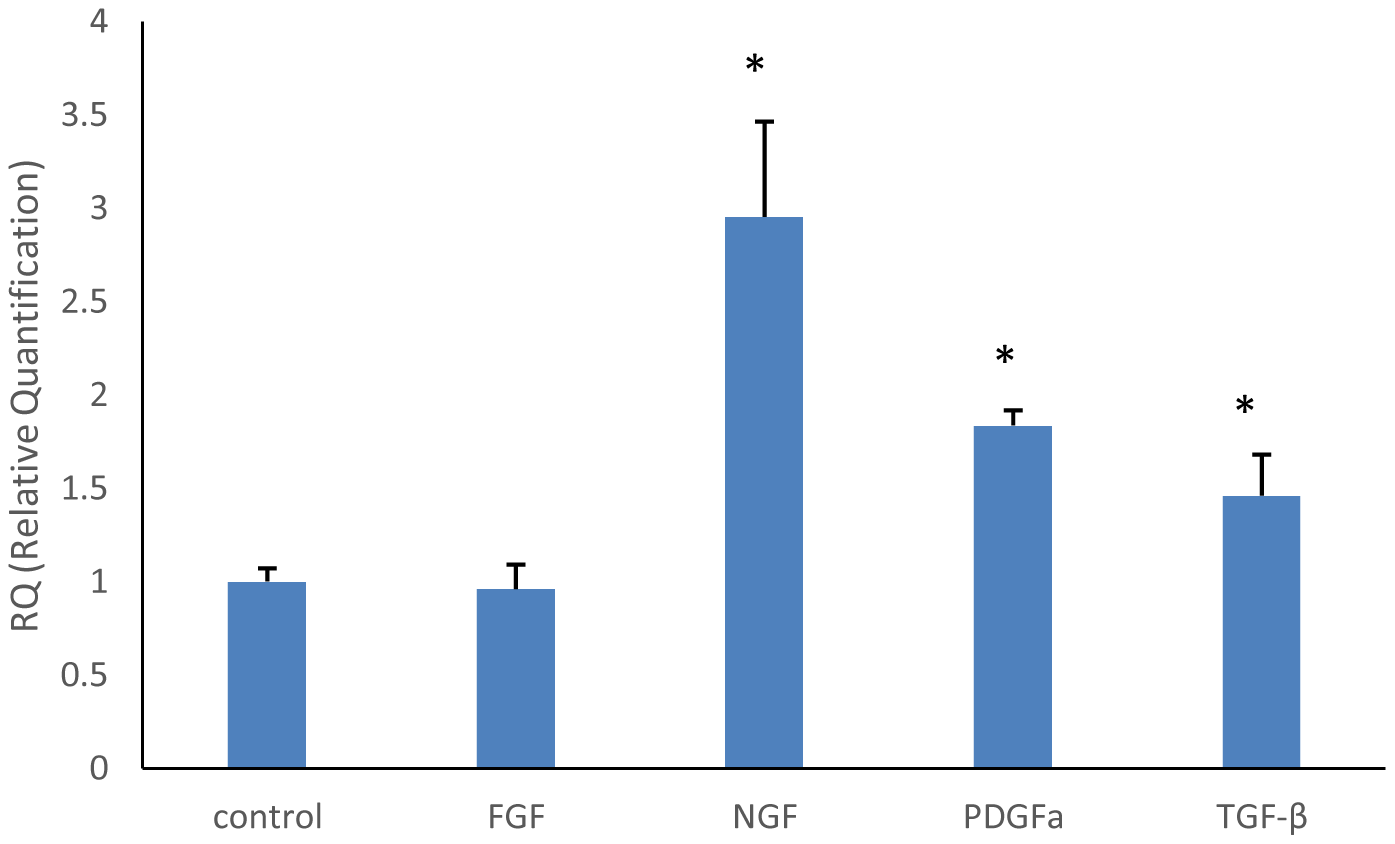
Changes in mRNA levels of FGF, NGF, PDGF, and TGF-β of rat sciatic nerve segments conditioned by IL-1β. ^*^ *p*<0.05 versus control.

Within distal portions of the transection gap zone we identified clusters of macrophages labeled with CD68 where the density of macrophage was increased as the frequency of ES was increased (Figure 4). Specially, a significantly higher density of macrophages was recruited into the nerve stumps in the ES-treated animals at 200 Hz compared to that at 0, 2, and 20 Hz of ES and the normal controls (P<0.05). Finally, it was found that the NGF, PDGF, and TGF-β-mRNA levels were significantly increased in the rat sciatic nerve segments (P<0.05) after addition of recombinant rat IL-1β (Figure 5).

## Discussion

Previous experimental studies have demonstrated evidence of the influence of ES on peripheral nerve regeneration using laboratory animals. Foecking et al. (2012) found ES administered immediately following a crush injury in rats could decrease the time for complete recovery from facial paralysis [11]. Teodori et al. (2011) found high-voltage ES could accelerate the functional recovery, potentiate the nerve fibers maturation and decrease macrophages and connective tissue area density after sciatic nerve crush injury in rats [12]. Similarly, Alrashdan et al. (2011) indicated low-intensity ES is an effective technique enhancing the sciatic nerve regeneration following a crush injury in rats [13]. In these studies, short crush lesions were used that the basal lamina in the injured nerve trunks could still be maintained, which is not observed in a transection injury [14]. Unlike these previous studies, the present work utilized the silicone rubber chamber model with a greater gap length for *in vivo* studies of peripheral nerve regeneration in diabetic rats. In the literature, it has been reported nerve regeneration process may be directly impaired by diabetes mellitus, caused by deficits in action of vasoactive neuropeptides such as CGRP, resulting in a relative ischemic state in regenerative microenvironment [15]. It may also delay the recruitment of macrophages to injury sites, slowing down the Wallerian degeneration following nerve injury [16]. In our previous study, we have found that application of brief ES could increase the action of CGRP and the number of macrophages in diabetic nerves following injury which may accelerate axonal re-growth and functional recovery [6]. Despite this knowledge, there is no experimental validation to confirm the influences of different frequencies of ES on nerve regeneration in diabetic animals.

In the present study, we found that the application of ES treatment, especially at 200 Hz, could significantly decrease the latency and increase the NCV as well as the amplitude and area of the MAPs of regenerated nerves as compared to the lower-frequency ES-treated groups. These results indicate that the transected nerves receiving the high-frequency of ES treatment had undergone better regeneration which had reinnervated the muscle fibers. These results are also supported by our morphometric data that a higher frequency of ES could increase the success rate of regenerated nerve cables in the bridging silicone rubber chambers. In addition, the high-frequency ES had also accelerated nerve fiber maturation, characterized by increased myelinated axon count. The way in which activity ameliorates diabetic neuropathy is not known. However, investigating the effects of ES can help in the understanding of how different frequencies of ES affect the regenerating nerve fibers in diabetic animals. Firstly, we found that elevating the frequency of ES could dramatically increase the cutaneous blood flow in the hindpaw ipsilateral to the injury of the diabetic rats. In the literature, it has been reported that increased blood flow velocity could be caused by muscle contraction induced by the ES treatment since the density of microvessels in the vascular bed can be increased in the contracted muscle [17], [18]. Investigators suggest that the changes in the blood vessel and local hemodynamics caused by ES may be mediated via a reflex arc, consisting of somatic afferent and autonomic efferent components [19] or via the adenosine, a vasodilator as well as a metabolite of muscle contraction [20]. As we know, adequate blood flow is vital to the viability and development of regenerating nerves. Therefore, these results suggest that external ES could induce a transient rise in peripheral perfusion which is beneficial to nerve regeneration in rats with diabetes. Secondly, we found that ES at higher frequencies, especially at 200 Hz showed enhanced effects on CGRP expression in the dorsal horn of the diabetic rats. Since CGRP has been recognized as a nerve regeneration-promoting peptide *in vivo* [21], it is conceivable that the elevated CGRP expression in the spines seen in the present study may be attributable to the fact that more injury-related signals derived from the diabetic nerves could be provoked after the ES treatments and retrogradely transported to neurons in the dorsal horn and subsequently trigger more cells to synthesize and release CGRP. Thirdly, we found that ES at higher frequencies could promote the invasion of macrophages into the endoneurium following nerve injury in diabetic animals. Apart from their role in removing myelin debris from the degenerating process, the macrophages and their released IL-1β were found in the study that they could also stimulate the secretion of NGF, PDGF, and TGF-β in dissected nerve segments, which could exert neurotrophic effects on regenerating nerve fibers [22]–[24]. The diabetic animals in the lower-frequency ES-treated groups seen in the present study showed a comparatively reduced number of macrophages in the distal sciatic nerve after injury may indicate that there could be a delay in Wallerian degeneration accompanied with less secretion of neurotrophic factors, resulting in impaired regeneration. The supplementary high-frequency ES treatments could potentially accelerate nerve degeneration process and promote macrophage invasion to release more neurotrophic factors leading to enhancement of the regenerative response of diabetics.

Though advancement in the degree of regeneration with increased frequency of ES was seen, there were still limitations in the present study should be addressed. For example, no statistically significant improvement was demonstrated in the morphological analysis. This may have been in part due to the small sample size, since a small increment of improvement was noted. As we know, great species variability could happen in the nerve regeneration processes, caution must be given not to extrapolate too much from animal studies. Besides, single methods of measuring peripheral nerve regeneration give only limited data, but by combining methods a better understanding of peripheral nerve regeneration is possible. Though the small sample size in the numbers of myelinated fibers in each group may not reflect practical correlations among regenerated nerves, however, in conjunction with noting improvements in the ultrastructural, the immunohistochemical, and the electrophysiological analyses as well as how the morphometric data clustered in the grouping comparisons, meaningful advantages still could be identified.

In conclusion, to the best of our knowledge, this is the first study of ES’s effects at different frequencies on peripheral nerve regeneration in diabetic rats. The information from this study provides a basis to consider using ES at higher frequencies as complementary treatments for diabetic patients with a severed peripheral nerve with a long gap to be repaired.
